# Effects of Bentonite on *p*-Methoxybenzyl Acetate: A Theoretical Model for Oligomerization via an Electrophilic-Substitution Mechanism

**DOI:** 10.3390/molecules16021761

**Published:** 2011-02-21

**Authors:** Manuel Salmón, Rene Miranda, Ines Nicolás-Vázquez, Yolanda Marina Vargas-Rodriguez, Julian Cruz-Borbolla, María Isabel Medrano, José Antonio Morales-Serna

**Affiliations:** 1 Instituto de Química de la Universidad Nacional Autónoma de México, Circuito Exterior, Ciudad Universitaria Coyoacán 04510, México, D.F., Mexico; E-Mail: morser@correo.unam.mx (J.A.M.-S); 2 Sección de Química Inorgánica, Fisicoquímica y Química Orgánica, Departamento de Ciencias Químicas, Facultad de Estudios Superiores Cuautitlán, Universidad Nacional Autónoma de México, Cuatitlán Izcalli, 54740, Estado de México, Mexico; E-Mails: mirruv@yahoo.com.mx (R.M.); ym_vargas@yahoo.com.mx (Y.M.V.-R.); 3 Área Académica de Química, Universidad Autónoma del Estado de Hidalgo, Unidad Universitaria, Km 4.5 Carretera Pachuca-Tulancingo, Mineral de Reforma, 42184, Hidalgo, Mexico; E-Mail: jcruzborbolla@yahoo.com.mx (J.C.-B.)

**Keywords:** bentonitic clay, *para*-methoxybenzyl acetate, oligomerization, electrophilic aromatic substitution, DFT calculations

## Abstract

Tonsil Actisil FF, a commercial bentonitic clay, promotes the formation of a series of electrophilic-aromatic-substitution products from *para*-methoxybenzyl acetate in carbon disulfide. The molecules obtained correspond to linear isomeric dimers, trimers, tetramers and a pentamer, according to their spectroscopic data. A clear indication of the title mechanistic pathway for the oligomerization growth was obtained from the analysis of a set of computational-chemistry calculations using the density-functional-theory level B3LYP/6-311++G(d,p). The corresponding conclusions were based on the computed dipole moments, the HOMO/LUMO distributions, and a natural-populations analysis of the studied molecules.

## 1. Introduction

Benzyltoluenes are of industrial interest due to their application as insulating oils in high-voltage electrical devices [1], in the production of termiticide emulsions that demonstrate good penetration and emulsion stability [2], and in the preparation of corrosion-protection products [3]. Our research group previously developed and reported a strategy to obtain oligomeric toluene compounds with solid acid catalysts using thermal energy and ultrasound [4]. Thus, when *ortho-* and *para*-benzyltoluenes were studied, the outcome of the oligomerization reactions was found to be dependent of the amount of catalyst used, reaction time, temperature and the presence of pyridine as a competitive inhibitor. The study was carried out using Tonsil Actisil FF (TAFF), a commercial bentonite clay that has long been employed by our research group as a Brønsted-Lowry and Lewis acid catalyst in different chemical reactions [5,6,7,8,9,10,11,12,13,14]. An electrophilic aromatic substitution pathway has been suggested for the oligomerization reaction [15], while a low toluene/BzCl ratio is necessary to obtain the oligomers [16,17].

In this context, we considered the possibility of carrying out the reaction of *para*-methoxybenzyl acetate in the presence of Tonsil Actisil FF to obtain oligomeric compounds, followed by a theoretical analysis that rationalizes the observed products and some of the key reaction steps. Previous studies carried out by our research group have established the importance of an electrophilic attack in similar reactions [18]. Despite the complete absence of experimental data on the structures of the transition states, calculated transition-state geometries are now commonplace thanks to the development of software that provides tools to propose transition states. Thus, the aim of this paper is to report on the conversion of *para*-methoxybenzyl acetate with TAFF, producing isomeric diphenylmethane and a series of linear oligomers with a progressive number of units. This is accompanied by computational-chemistry calculations performed to establish a validated mechanistic pathway to explain the growth of the obtained linear oligomers.

## 2. Results and Discussion

**Figure 1:**
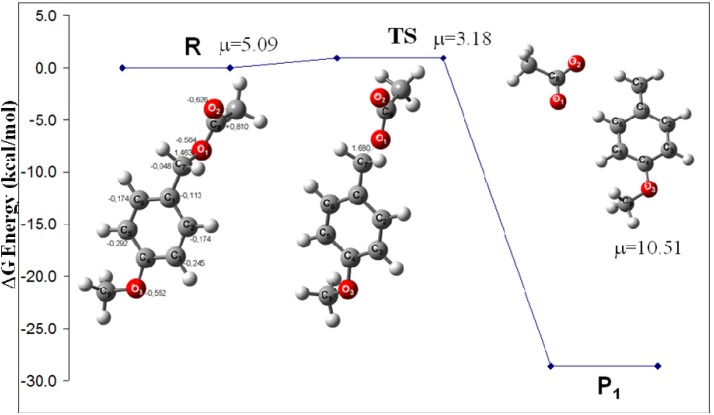
B3LYP/6-311++G(d,p) energy profile for the carbenium ion formation.

To investigate the novel catalytic capabilities of TAFF, this work studied the transformation of *para*-methoxybenzyl acetate (**1**). Despite the Lewis and Brønsted-Lowry acid character of the studied clay, its acidity is very low in comparison with sulfuric or triflic acids [19]. Thus, the phenylmethanes **2**-**4** and linear oligomers **5**-**8** were obtained when the reaction was carried out in carbon disulfide at room temperature. However, when the temperature was increased to 46 °C, the linear oligomers **9 **and **10 **were obtained. The structure of each isolated compound is displayed in Figure 1, where it can be seen that the phenylmethanes **2** and **3** are *ortho*-substituted with respect to MeO, an electron-donating group, whereas **4** is formed by an *ipso*-substitution. All other molecules are symmetrically substituted linear benzylic oligomers formed through an electrophilic aromatic substitution (EAS) with three, four or five linked aromatic units. In the oligomers **2**, **5** and **8**, the acetyl unit remains present, but it is absent in **3** and **7**, yielding a methyl group, as previously seen in pentamethylbenzyl cation reduction [20] by the addition of a hydride provided by the clay [21]. However, this process could results from a catalytic transfer hydrogenation from an organic donor-molecule species to any of the variety of organic acceptors formed in the reaction medium [22]. Trimer **6** is the only isolated compound possessing a benzylic OH group, which is probably formed after the acyl hydrolysis of **5**. Finally, when the temperature is increased, the promotion of the longer linear oligomers **9 **and **10** is obtained by further condensation with other *para*-methoxybenzylic carbenium ions or by an *ipso* substitution.

**Scheme 1:**
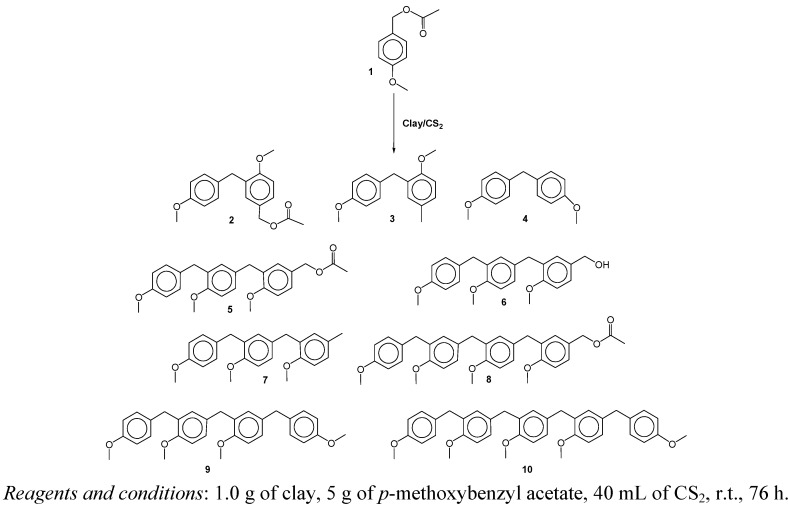
Oligomerization reaction products.

**Figure 2:**
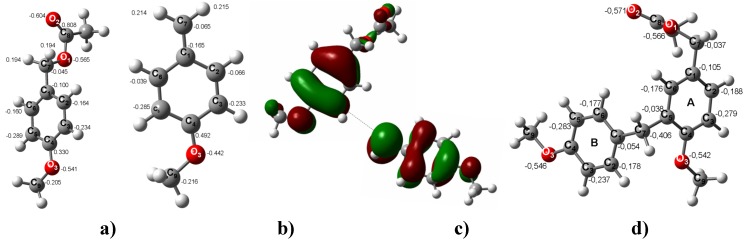
**a)** Neutral species of *para*-methoxybenzyl acetate. **b)** The carbenium ion of *para*-methoxybenzylic acetate. **c)** The HOMO of *para*-methoxybenzyl acetate and the LUMO of the *para*-methoxybenzyl carbenium ion (ΔE_HOMO-LUMO_ = 1.69 eV). **d)** The optimized B3LYP/6-311++G(d,p) geometries for **2**. The calculated NPA charges are reported in **e^-^**.

**Figure 3:**
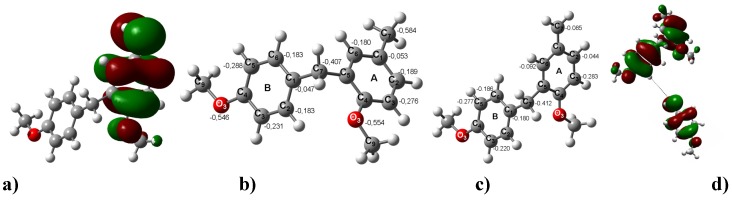
**a)**The LUMO of the dimeric carbenium ion that yields **3**. **b)** The optimized B3LYP/6-311++G(d,p) geometries for **3**. The calculated NPA charges are reported in **e^-^**. **c)** The carbenium dimer. **d)** The interaction between the LUMO of the charged carbenium dimer with the HOMO of **3** to yield the corresponding trimer **7**.

Two possibilities were considered to explain the EAS mechanism of the *para*-methoxybenzyl acetate reactions yielding **2**-**10** (Scheme 1) via the *para*-methoxybenzyl carbenium ion. The clay may provide protons or electrons to the neutral molecule to assist the carbocation induction at the beginning of the process. To analyze this possibility, the benzylic CH_2_-OAc bond distance in the monomer was computed with a charge of –1 and a length of 1.680 Å (adding one electron) and with a charge of +1 and a length of 1.418 Å (removing an electron) (Figure 1). It is important to note that, in the neutral molecule, the CH_2_-OAc bond distance is 1.463 Å. With these bond lengths, the clay may provide an electron to enlarge the CH_2_-OAc bond, easing the corresponding rupture to generate an electrophilic species. In this sense, **2** must be formed when this carbenium ion, with a dipole moment (DM) of 10.51 D and located in the plane of the benzene ring, interacts with the 3.17 D DM of a neutral species. In this way, the interaction between both species with an *ortho* position to the MeO group on the benzene ring will be favored (Figure 2). Additionally, this mechanism may suggest that the formation of **3** is obtained from the dimeric carbenium ion (Figure 3), which is reduced by a hydride provided by the clay, yielding the corresponding methyl group [20].

**Figure 4:**
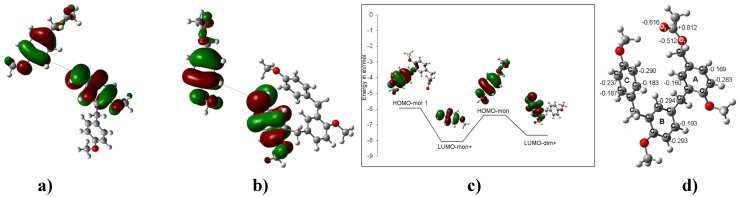
**a)** The interaction between the LUMO of the charged carbenium dimer with the HOMO of the original substrate to yield the corresponding oligomer **5**. **b)** The interaction between the LUMO of the charged carbenium trimer with the HOMO of the original substrate to yield the corresponding oligomer **8**. **c)** Two possible routes to obtain **5**. The energy difference between the HOMO of the neutral species and the LUMO of the dimer carbenium is ΔE_HOMO-LUMO_ = 1.28 eV; therefore, this interaction is more feasible. **d)** The optimized B3LYP/6-311++G(d,p) geometries for **5**. The calculated NPA charges are reported in **e^-^**.

Consequently, all compounds possessing a benzylic-ester substituent group, after dimerization, are able to continue the chain enlongation assisted by the corresponding benzylic carbenium ion. Thus, the oligomers **5** and **8** may be formed by two possible routes. The first route implicates an interaction between the charged carbenium dimer **2** or a charged carbenium trimer **5** with the original substrate (basic unit) to yield the corresponding trimer **6** and tetramer **8**. This model resembles the process of formation of **2** (Figure 4).

**Figure 5:**
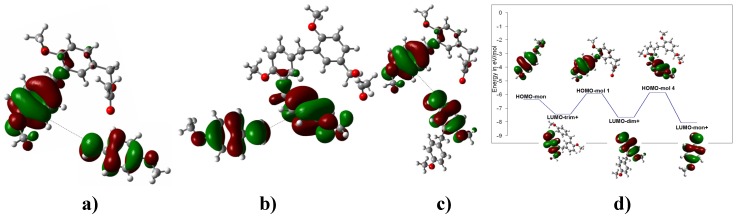
**a)** The oligomer **5** is obtained from the benzylic carbenium monomer and the dimer **2**. **b)** The HOMO of **5** and the LUMO of methoxybenzyl carbenium ion that yield tetramer **8**. **c)** The HOMO of **2** and the LUMO of the charged carbenium dimer that yield **8**. **d)** According to the graph, three possible routes could lead to **8**. The energy difference between the HOMO of the neutral species and the LUMO of the trimer carbenium is ΔE_HOMO-LUMO_ = 1.06 eV; therefore, this interaction is more feasible.

**Figure 6:**
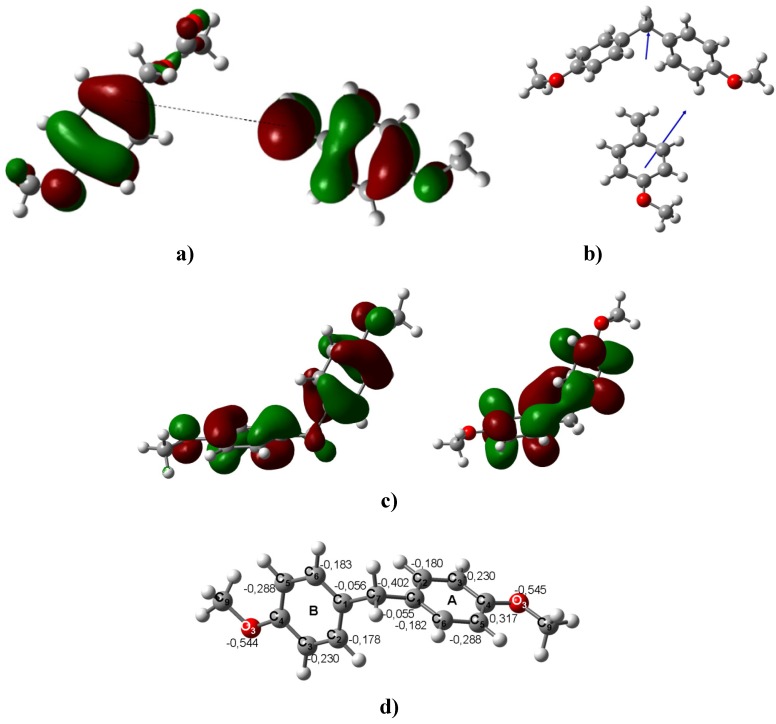
**a)** The HOMO of *para*-methoxybenzyl acetate and the LUMO of the methoxybenzyl carbenium ion that yield oligomer **4**. **b)** The DM of the carbenium ion must be aligned towards the methylene group of **4**. This centered orientation prevents any further substitution under the reported experimental conditions. **c)** The HOMO and LUMO of **4**. **d)** The optimized B3LYP/6-311++G(d,p) geometries for **2**. The calculated NPA charges are reported in **e^-^**.

The oligomer **5** may be obtained from the benzylic carbenium monomer and the dimer **2** instead of via the second pathway (Figure 5). The same condensation is suggested to explain the formation of tetramer **8**; it is the primary interaction that occurs between the two DMs and the carbenium ion oriented towards the benzene ring with the major HOMO distributions. Thus, this interaction is towards any of the C atoms located *ortho* to the MeO group. It is also worth mentioning that these atoms have a natural atomic charge of -0.290 e^-^, the largest negative value in this ring. The HOMO distributions can be observed in the B ring. The C atoms located in *ortho* to the MeO group have natural atomic charges of -0.293 and -0.294; therefore, these sites could interact with the LUMO of the carbenium ion.

**Figure 7:**
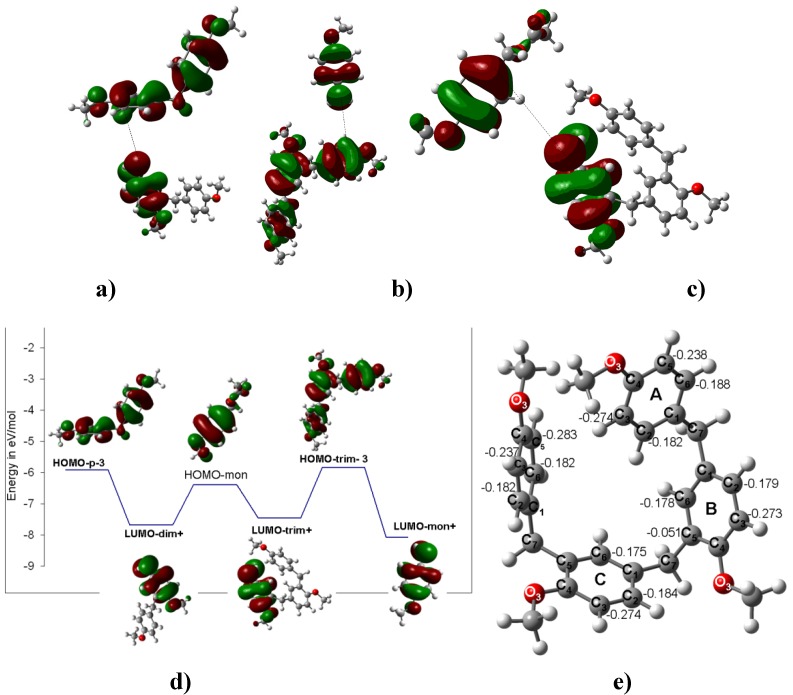
**a) **Interaction between the LUMO of the charged carbenium dimer and the HOMO of **4** to yield the corresponding trimer **9**. **b)** The interaction between the LUMO of methoxybenzyl carbenium ion and the HOMO of the trimer of **4** to yield the corresponding tetramer **9**. **c)** The interaction between the LUMO of the charged carbenium trimer and the HOMO of the monomer. **d)** Three possible routes to obtain **9**. The energy difference between the HOMO of the neutral species and the LUMO of the trimer carbenium is ΔE_HOMO-LUMO_ = 1.76 eV; therefore, this interaction is more feasible. **e)** Optimized B3LYP/6-311++G(d,p) geometries for **9**. The calculated NPA charges are reported in **e^-^**.

**Figure 8:**
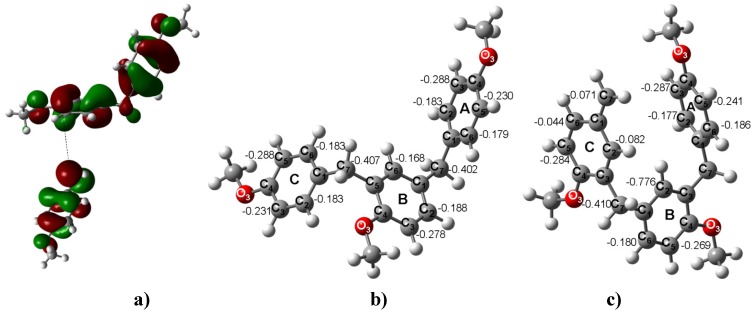
**a)** The interaction between the LUMO of the methoxybenzyl carbenium ion with the HOMO of **4** to yield the corresponding trimer, **4a**, as an intermediary of **9.b)** Optimized B3LYP/6-311++G(d,p) geometries for **4a**. The calculated NPA charges are reported in **e^-^**. c) Carbenium trimer of **4a**.

**Figure 9:**
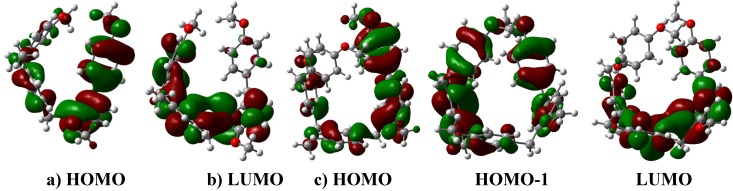
**a)** The HOMO and LUMO of **9**. **b)** The HOMO, HOMO-1 and LUMO of **10**.

**Figure 10:**
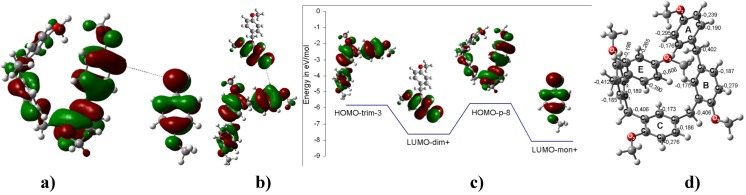
**a)** Interaction between the LUMO of the methoxybenzyl carbenium ion and the HOMO of **9** to yield the corresponding pentamer **10**. **b)** The interaction between the LUMO of the charged carbenium dimer and the HOMO of the trimer of **4** to yield the corresponding pentamer **10**. **c) and d)** Two possible routes to obtain **10**. The energy difference between the HOMO of the trimer neutral species and the LUMO of the dimer carbenium is ΔE_HOMO-LUMO_ = 1.82 eV; therefore, this interaction is more feasible. **e)** The optimized B3LYP/6-311++G(d,p) geometries for **10.** The calculated NPA charges are reported in **e^-^**.

Moreover, the inhibition of the EAS was clear in **4**. Its computed DM is 0.74 D and points outwards from the methylene group. The computed HOMO distribution is widespread in an equivalent form over both benzene rings (Figure 6). It is also important to mention that the DM of the carbenium ion must be aligned towards the methylene group. This centered orientation and the even distribution of the HOMO and LUMO will prevent any further substitution under the reported experimental conditions. The *ipso* substitution plays an interesting role in the oligomerization control by blocking one side of the molecule, such as in **9** and **10** (Figure 7, Figure 8 and Figure 9). Thus, **9** and **10**, which have DMs of 1.99 D and 2.25 D, respectively, and their HOMOs and LUMOs (Figure 10) located on opposite ends of the chain, favor substitution, and the chain elongation proceeds step-by-step by condensing a carbenium ion with the benzene ring that holds the HOMO orbitals. The reaction will be directed to the *ortho*-methoxy carbons with the largest negative charge in the ring (Figure 7).

It is convenient to note that, to classify the catalytic hydrolysis of ester molecules, organic-chemistry textbooks state that the reaction precedes via AAc2 under acidic conditions and BAc2 under basic conditions [23]. The above notation indicates that the O-R bond is not commonly broken [24,25,26,27,28,29]. Under acid catalysis, an alternative but less common pathway involves the alkyl-oxygen cleavage in which an acyloxy group or its conjugated acid is the leaving group; thus, an AAl_1_ mechanism occurs if the alkyl substituent comes off as a stable carbenium ion. In this particular sense, it is already known that benzylic acetates are hydrolyzed by the AAc_2_ mechanism in dilute acid, whereas the mechanism changes to AAl_1_ in concentrated acid [30,31,32,33,34]. Additionally, little is known about acetylated benzylic alcohols as electrophiles in the EAS reaction. However, it is worth mentioning that the driving force for this process must be the catalytic acidic promotion of an AAl_1_ mechanism, yielding such interesting intermediates as the *para*-methoxybenzylic carbenium ion. Finally, it is important to note that, to the best of our knowledge, this work is the first example of a proposed AAl_1_ pathway when bentonitic clay is used as a heterogeneous catalyst in an EAS reaction, as supported by theoretical calculations.

**Scheme 2:**
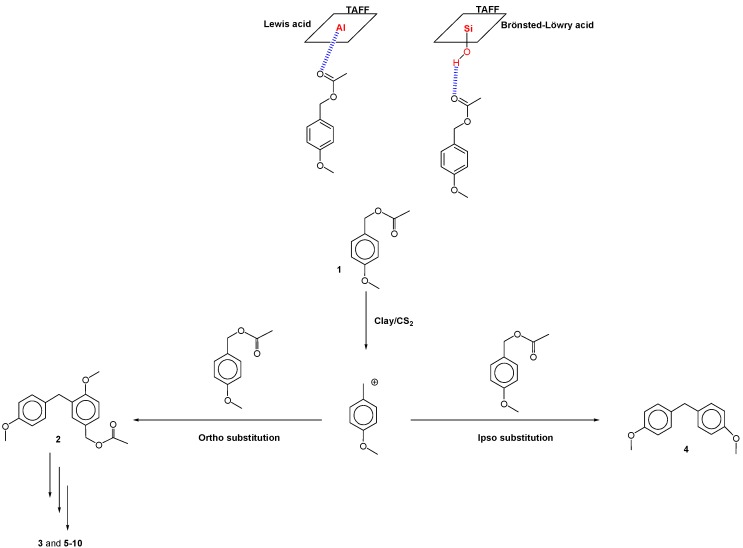
Proposed reaction mechanism.

Finally, from the mechanistic point of view, the catalytic action of TAFF should enhance the electrophilic character of the carbonylic substrates, facilitating the formation of *para*-methoxybenzyl carbonium ion. The ion is the key intermediate to understand the formation of the products. The interaction between TAFF and **1** might be due to the protonated and unprotonated active sites corresponding to the acidic Brönsted-Löwry and Lewis character of the clay (Scheme 2).

## 3. Experimental

### 3.1. General

Tonsil Actisil FF, a cheap (US $1.30/kg) commercial Mexican bentonitic clay, is readily available from Tonsil Mexicana S. A. de C. V. (México City, México). Examined with X-ray fluorescence, this clay was shown to have the following composition (in %): SiO_2_, 74.5; Al_2_O_3_, 9.3; MgO, 0.4; Fe_2_O_3_, 1.3; CaO, 4.0; K_2_O, 0.4; TiO_2_, 0.4; H_2_O, 9.7. X-ray thermodiffractograms show that the laminar structure is unstable above 150 °C. Quartz and cristobalite are also important components in the clay composition, as observed by X-ray diffraction. The corresponding BET surface area is 198.718 m^2^ g^−1^, and the pore volume and average pore diameter are 32.04 × 10^−2^ cm^3^ g^−1^ and 77.8 Å, respectively. It is worth mentioning that a detailed characterization of the clay (^29^Si and ^27^Al MAS-NMR, SEM, IR-Py, DTA, TG, and Ho) has already been performed and reported by our research group [12]. The particle size is 325 mesh. The organic reagent 4-methoxybenzylacetate was prepared by the acetylation of 4-methoxy-benzyl alcohol (Merck) with pyridine and acetyl chloride and was further purified by SiO_2_ column chromatography using *n*-C_6_H_14_/EtOAc as the eluent. IR spectra were recorded on a Nicolet FTIR-5SX spectrometer. The ^1^H- and ^13^C-NMR spectra were measured in CDCl_3_ solution with a Varian Unity 300 (operating at 300 MHz and 75 MHz, respectively) using tetramethylsilane as an internal standard. Mass spectra were obtained with a JEOL JMS AX505HA mass spectrometer. For thin-layer chromatographic (TLC) analyses, Merck precoated TLC plates (silica gel 60 F_254_, 0.25 mm, Art 5715) were used.

### 3.2. Oligomerization of p-methoxybenzyl acetate with Tonsil

The typical procedure at room temperature is as follows: a suspension of *p*-methoxybenzyl acetate (1, 5.0 g), carbon disulfide (40 mL) and the bentonitic earth (1.0 g) was vigorously stirred at room temperature until disappearance of the starting material, which occurred after 76 h. The reaction was conveniently monitored by TLC. The clay was eliminated by filtration through Celite and washed with ethyl acetate (3 × 10 mL). The combined filtrates were dried on anhydrous Na_2_SO_4_, and the solvent was eliminated under reduced pressure. The residue was subjected to chromatography on a silica-gel column using *n*-C_6_H_14_/EtOAc as the eluent, affording phenylmethanes **2**-**4** and linear oligomers **5**-**8**. The typical procedure at reflux is identical to the above procedure, but the chromatography afforded in that case the linear oligomers **9** and **10**.

*3-(4-Methoxybenzyl)-4-methoxybenzyl acetate* (**2**). Syrupy yellow oil, isolated in 3.8% yield, (C_18_H_20_O_4_). IR (film, cm^−1^): 3,007, 2,953, 2,836, 1,735 (C=O), 1,464, 1,263, and 1,032 (C-O). ^1^H-NMR δ: 2.05 (3H, s, CH_3_), 3.77 (3H, s, CH_3_-O), 3.78 (3H, s, CH_3_-O), 3.90 (2H, s, Ar-CH_2_-Ar), 4.98 (2H, s, CH_2_O), 6.80–6.85 (3H, m, Ar), 7.05–7.21 (4H, m, Ar), ^13^C-NMR δ: 21 (CH_3_), 35 (CH_2_), 56 (2C, CH_3_O), 66 (CH_2_O), 110,114, 128, 134, 156 and 158 (C, Ar). 171 (C=O). MS *m/z*: 300 [M^+^, 100%], 241 [M-AcO]^+^ (54), 225 (17), 209 (16), 137 (18), 121(50), 91(10).

*1-(2-Methoxy-5-methylbenzyl)-4-methoxybenzene* (**3**). White needles m.p. 64–66 °C, 0.42% (C_16_H_18_O_2_). IR (CDCl_3_ cm^−1^): 3,032, 2,919, 2,835, 1,466, 1,442, (CH_2_, CH_3_) 1,250, 1,032 (C-O). ^1^H-NMR δ: 2.23 (3H, s, CH_3_), 3.77 (3H, s, CH_3_-O), 3.78 (3H,s, CH_3_O), 3.88 (2H, s, CH_2_) 6.7–6.9 (3H, m, Ar), 6.7–7.11, (4H, d, Ar, *J* = 8.7, 8.4 Hz A_2_B_2_); ^13^C-NMR δ: 22 (CH_3_), 35(CH_2_), 56 (CH_3_O), 111, 114, 127, 130, 131.5 (C, Ar), 171 (C=O). MS *m/z*: 242 [M^+^, 100%], 227 [M-Me]^+^ (35), 211 [M-OMe]^+^ (43), 195 (13), 174 (17), 121 (46), 91 (15)

*Bis(4-methoxyphenyl)methane* (**4**). Syrupy yellow oil 0.3% (C_15_H_16_O_2_). IR (CHCl_3_, film, cm^−1^): 3,005, 2,920, 2,839, 1,511, 1,457, 1,435, 1,252, 1,027. ^1^H-NMR δ: 3.78 (6H, s, CH_3_O), 3.86 (2H, s, CH_2_), 6.8 (4H, d, *J* = 9 Hz, Ar), 7.05 (4H, d, *J* = 8.4 Hz, Ar) A_2_B_2_. ^13^C-NMR δ: 40 (CH_2_), 56 (2C, CH_3_O), 114 (4C, Ar *o*-H_3_CO-C-CH), 130 (4C, Ar, *m*-H_3_CO-C-CH-CH) 134 (2C Ar, *ipso*), 158 (2C, Ar, H_3_CO-C). MS *m/z*: 228 [M^+^, 100%], 227 [M-H]^+^ (55), 197 [M-OMe]^+^ (85), 121 (28), 114 (12), 91 (7), 77 (4).

*3-(3-(4-Methoxybenzyl)-4-methoxybenzyl)-4-methoxybenzyl acetate* (**5**). Syrupy yellow oil 1.3% (C_26_H_28_O_5_). IR (CHCl_3_, film, cm^−1^): 2,835, 1,736, (C=O) 1,463, 1,441, 1,248, 1,033. ^1^H-NMR δ: 2.05 (3H, s, CH_3_), 3.77 (9H, m, CH_3_O), 3.85, 3.88 (4H, s, 2CH_2_, 4.98 (2H, s, CH_2_O), 6.75–7.20 (10 H, m, Ar). ^13^C-NMR δ: 21 (CH_3_), 35 (CH_2_), 56 (CH_3_O) 66 (CH_2_O) 110, 114, 128–134, 156–158 (Ar), 171 (C=O): MS *m/z*: 420 [M^+^, 37%], 360 [M-AcOH]^+^ (100), 345 [M-AcOH-Me]^+^ (56), 299 (10), 239 (12), 227 (10), 165 (6), 121 (90), 91 (6).

*(3-(3-(4-Methoxybenzyl)-4-methoxybenzyl)-4-methoxyphenyl)methanol* (**6**). Syrupy yellow oil 0.0075% (C_24_H_26_O_4_). IR (CHCl_3_, film, cm^−1^): 3,425 (OH), 2,850, 2,928, 1,502, 1,247, 1,033, 1,463, 835. ^1^H-NMR δ: 3.77 (9H, s, CH_3_O), 3.84 (2H, s, CH_2_), 3.86 (2H, s, CH_2_), 4.54 (2H, s, CH_2_), 6.7–7.18 (10H, m, Ar); ^13^C-NMR δ: 30 (CH_2_), 35 (CH_3_O) 65 (CH_2_O) 110, 114, 126–132 (Ar); MS *m/z* 378 [M^+^, 75%], 360 [M-H_2_O]^+^ (16), 345 [M- H_2_O -Me]^+ ^(15), 257 (46), 227 (42), 195 (12), 149 (15), 121 (100), 97 (11), 81 (16), 69 (34), 41 (25).

*4-(2-Methoxy-5-methylbenzyl)-2-(4-methoxybenzyl)-1-methoxybenzene* (**7**). Syrupy yellow oil 0.58% (C_24_H_26_O_4_). IR (CHCl_3_, film, cm^−1^): 3,032, 2,929, 2,833, 1,520, 1,463, 1,461, 1,260, 1,035. ^1^H-NMR δ: 2.21 (3H, s, CH_3_), 3.73 (3H, s, CH_3_O), 3.77 (3H, s, CH_3_O), 3.81 (2H, s, CH_2_), 3.86 (2H, s, CH_2_), 6.71–7.12 (10H, m, Ar); ^13^C-NMR δ: 21 (CH_3_), 35 (CH_2_) 56 (CH_3_O), 110, 114, 127, 130, 131, (Ar); MS *m/z* 362 [M^+^, 100%], 347 [M-Me]^+ ^(8), 331 [M-OMe]^+^(10), 241 (77), 227 (41), 195 (6), 181 (7), 121 (95), 105 (10), 91 (6).

*3-(3-(3-(4-Methoxybenzyl)-4-methoxybenzyl)-4-methoxybenzyl)-4-methoxybenzyl acetate* (**8**). Syrupy yellow oil 0.037% (C_34_H_36_O_6_); IR (CHCl_3_, film, cm^−1^): 2,925, 2,835, 1,736, (C=O), 1,619, 1,254, 1,033 cm^−1^. ^1^H-NMR δ: 2.04 (3H, s, CH_3_), 3.73–3.77 (12H, s, CH_3_O), 3.80–3.85 (6H, s, CH_2_), 4.95 (2H, s, CH_2_), 6.7–7.25 (12H, m, Ar); ^13^C-NMR δ: 21 (CH_3_), 35, 30, (CH_2_) 56 (CH_3_O), 67 (CH_2_O), 171 (C=O), 110, 114, 127-130, (Ar); MS *m/z* 480 [M^+^, 100%], 465 [M-Me]^+ ^(23), 449 [M-OMe]^+^(11), 345 (6), 240 (22), 121 (83), 91 (4), 57 (6).

*1-(5-(5-(4-Methoxybenzyl)-2-methoxybenzyl)-2-methoxybenzyl)-4-methoxybenzene* (**9**). Syrupy yellow oil 0.39% (C_34_H_36_O_6_); IR (CHCl_3_, film, cm^−1^): 2,928, 2,837, 1,611, 1,246, 1,043. ^1^H-NMR δ: 3.73 (3H, s, CH_2_O), 3.77 (6H, s, CH_3_O), 3.79 (4H, s, CH_2_), 3.86 (2H, s, CH_2_), 6.7–7.12 (14H, m, Ar); ^13^C-NMR δ: 35, 41 (CH_2_), 55 (CH_2_O), 110, 114, 126–134, (Ar); MS *m/z* 480 [M^+^, 100 %], 437 [M-OMe]^+ ^(7), 347(32), 241(10), 227(31), 121(50).

*2-(3-(4-Methoxybenzyl)-4-methoxybenzyl)-4-(5-(4-methoxybenzyl)-2-methoxybenzyl)-1-ethoxybenzene* (**10**). Syrupy yellow oil 0.94% (C_42_H_44_O_7_); IR (CHCl_3_, film, cm^−1^): 2,936, 2,834, 1,736, (C=O), 1,258, 1,033. ^1^H-NMR δ: 2.10 (3H, s, CH_3_), 371 (9H, s, CH_3_O), 3.75 (6H, s, CH_3_O), 3.79 (4H, s, CH_2_), 3.82 (2H, s, CH_2_), 3.84 (2H, s, CH_2_), 4.90 2H, s, CH_2_O), 6.7–7.25(16H, Ar); ^13^C-NMR δ: 22 (CH_3_), 35 (CH_2_), 55 (CH_3_O), 66 (CH_2_O), 110, 113, 127–134, 155, 158, (16H, Ar); MS *m/z* 660 [M^+^, 5%], 600 [M-AcOH] (100), 479 (55), 467 (11), 368 (22), 300 (11), 241 (23), 135 (22), 121 (70), 69 (26), 57 (39).

### 3.3. Theoretical calculations

The geometries and electronic structures of all compounds were determined by calculations carried out in the spin-restricted and spin-unrestricted formalisms for closed-shell and open-shell systems, respectively. In addition, the corresponding geometry optimizations were performed by electronic-structure approaches using density-functional theory (DFT) and specifically the functional B3LYP [35,36]. This functional is Becke´s three-parameter functional in which the correlation functional is provided by the Lee, Yang, and Parr expression (LYP) functional. B3LYP proved to be a suitable method because it includes the electron-correlation effects to some extent. In other words, this scheme was used in conjunction with the split-valence polarized basis set and diffuses, 6-311++G(d,p) [37,38,39,40], as implemented in Gaussian 03 [41].

The located transition state (TS) was confirmed through the analysis of vibration frequencies, which are single imaginary frequencies, defined along the reaction coordinate presented by such states. The optimized structures were confirmed as local minima by estimating their normal vibrations and by the absence of imaginary frequencies. The natural atomic charges, dipole moment and HOMO (highest occupied molecular orbital) and LUMO (lowest unoccupied molecular orbital) were used to analyze the electrophilic or nucleophilic character involved in the reactivity of the para-methoxybenzyl acetate molecule. In addition, these properties were determined for the equilibrium geometries. The structures and the properties were visualized with the GaussView package coupled to Gaussian 03. The natural-population analysis (NPA) was developed to calculate the atomic charges of molecular wave functions in general atomic-orbital basis sets. NPA is an alternative to conventional Mulliken population analysis and exhibits improved numerical stability [42,43]. This method was used to determine the charge of the studied molecules. The NBO 3.1 program was used as implemented in Gaussian 09 at the B3LYP theoretical level. The NBO analysis transforms the canonical delocalized Hartree–Fock (HF) MOs into localized orbitals that are closely tied to chemical-bonding concepts. This process involves the sequential transformation of non-orthogonal atomic orbitals (AOs) into sets of NAOs, NHOs, and NBOs. Each of these localized basis sets is complete and describes the wave functions in the most economical method because electron density and other properties are described by the minimal amount of filled orbitals in the most rapidly convergent way.

## 4. Conclusions

The oligomerization of *para*-methoxybenzyl acetate with Tonsil Actisil FF (TAFF), a commercial bentonite clay, provided isomeric diphenylmethane and linear oligomers with a progressive number of units. This work is the first example in which an AAl_1_ pathway is proposed when bentonitic clay is used as a heterogeneous catalyst in an EAS reaction. The corresponding conclusion was based on the computed dipole moments, the HOMO/LUMO distributions and a natural-populations analysis of the studied molecules.
